# Atlanta Academy of Medicine

**Published:** 1873-01

**Authors:** Jas. B. Baird

**Affiliations:** Reporter


### ATLANTA ACADEMY OF MEDICINE.

JAS. B. BAIRD, M.D., REPORTER.

[Extract from Minutes, July 29, 1872—Dr. Jno. G. Westmoreland, Vice-President, in the Chair.]

Dr. J. Stainback Wilson reported a case, supposed to be cerebro-spinal meningitis, in a girl nine years old. Had a chill on Thursday morning, followed by raving delirium during the day and following night. Saw her first the next day, Friday, in the forenoon. She had a violent convulsion early Friday morning. Her mother stated that she had a very high fever the day and night before, which had not disappeared on Friday. There was no rigidity of any muscle; she was excited; skin cool; was in constant motion, rolling and tossing from one side of the bed to the other, requiring at times to be restraine d; some swelling of the right elbow joint was observed; a very indistinct eruption was found over her body upon close inspection. Her mother represented that it was much more distinct and resembled flea bites the day before. Nausea was a prominent symptom throughout. She died at one o'clock Friday night.

Dr. Love remarked that it resembled some cases of malarial fever, with an extraordinary amount of congestion. Did not regard the fact of her being insensible as proof of meningitis.

Dr. Wilson replied that she had not been subjected to malarial influences.

Dr. Logan inquired if any member of the Academy had been accustomed to meet with rheumatism of the vertebral column simulating spinal irritation? Has now a case under treatment in which he feared serious organic disease of the spinal cord; but now regards it a clear case of rheumatism of vertebral column.

Dr. W. F. Westmoreland thought that we might have rheumatic deposits in the joints of the spine as well as any other joints.

Dr. Logan stated that the patient referred to was a lady, who had been an invalid for eighteen months, with supposed ovarian disease. She recently came to this city and placed herself under his care. She then suffered intense pain in various parts of her body. She subsequently had a distinct chill every day. There was considerable prolapsus, and engorgement and enlargement of the uterus; a slight enlargement, with severe pain and tenderness in one ovarian region; also pain and tenderness over the whole length of the spine, especially in the lumbar region. At this time there was considerable tenderness about some of the joints. Thought the spinal disturbance might be malarial, and put her on quinine, arsenic, strychnia, etc. After using this treatment for several days with benefit, it occurred to him that she was suffering from rheumatism. She was at once placed upon appropriate treatment, and for several days past has manifested decided improvement. He has been more disposed of late to look for rheumatism in the internal organs. He met with a case recently in the lungs which had been mistaken for tuberculosis. A gentleman came to this city from the north five or six months ago by the advice of his physician, thinking that he had tuberculosis. He had had several hemorrhages from the lungs; his case had been pronounced hopeless by his physician, and by his advice he was taking large quantities of alcoholic stimulants daily. He was very much depressed in spirits. He made a thorough examination of his case, and no evidence of tuberculosis was discovered. From some obscure indications, Dr. Logan suspected that he was suffering from rheumatism of the lungs, and advised the immediate discontinuance of the alcoholic liquors. He was soon afterwards attacked with acute articular rheumatism, from which he has recovered, and is now in better health than for five years past. The Doctor was led to study this subject by observing his own case. He is subject to rheumatism in almost every part of his body; sometimes it appears in his scalp, accompanied with a sensation of giddiness, followed by palpitation of the heart, evidently the result of subacute rheumatism of th t organ, joints, etc.; is satisfied that many obscure internal diseases of a rheumatic character are never discovered, which might be relieved by proper treatment.

Dr. W. F. Westmoreland recalled a case which occurred several years ago, evidently a case of rheumatic pneumonia; a stout negro man, subject to articular rheumatism, was attacked with pneumonia, presenting the symptoms and physical signs of pneumonia unmistakably. On the third day he was seized with violent pain in the knee joint; in less than twelve hours every sign of pneumonia had disappeared, and general articular rheumatism was developed.

Dr. Love believes that rheumatism of the gravid uterus will produce premature labor; has seen the crystalline deposit in the placenta as large as millet seed, in women who were subject to attacks of rheumatism.

Dr. Hughes, of ———, who was present, in response to a call from the chairman, reported the following anomalous case:

He was recently called to a woman in labor; she had been married six months; four weeks before was thrown from the back of a mule; which fall occasioned the premature labor. The pains came on and the head of the child presented, but would recede when the pains ceased. He was unable to deliver the child, and was at a loss to account for the unusual phenomenon. Upon examination he found the fœtus to be dead; he then introduced a knife into its abdomen and two gallons of water escaped; delivery was then affected. The fœtus was poorly developed, and the bony structure was in a softened, gelatinous condition.

---

August 12, 1872.

The President, Dr. Logan, presiding.

Dr. John G. Westmoreland reported a case, first seen by him three weeks ago. He regarded it at first as a case of remittent fever, and adopted the usual treatment for the disease—quinine, etc. Soon a redness of the tongue was noticed and diarrhœa occurred. Typhoid fever was then diagnosed. The tongue is now pale, and the diarrhœa has been arrested; considers the case convalescent. A decided pain in the occipital region was observed in the commencement of the disease.

His treatment was, remedies to arrest vomiting, a blister to the nape of the neck, fluid food, an opiate three times a day, alcoholic stimulants, mucilaginous drinks and astringents.

The President inquired what was his object in giving opium?

Dr. John G. Westmoreland replied, that he gave alcohol and opium to keep up a necessary vigor of the brain to admit of recovery; also to allay the accidental irritation of the bronchia and alimentary canal.

Dr. W. F. Westmoreland asked why he gave mucilaginous drinks?

Dr. John G. Westmoreland—gives it to soothe the inflamed mucous membrane, over which it passes; considers it a valuable *mechanical* remedy.

Dr. W. F. Westmoreland—thinks that it is assimilated, and does not pass through the ileum as gum arabic.

Dr. John Stainback Wilson desired to know the relation existing between the occipita-vertebral tenderness, referred to by Dr. Westmoreland, and typhoid fever?

Dr. John G. Westmoreland responded by saying that he looked upon that symptom as almost pathognomonic; it enables him to distinguish, in the early stages, between typhoid and remittent fever. In remittent fever the tenderness is spinal.

The President, Dr.. Logan, remarked, that while opium, occasionally used, might sustain the patient, it seemed to him that there was risk of exhaustion of the brain in continuing its use regularly for a considerable time. Thinks he has seen exhaustion due to it; considers it a questionable practice; its first action is to stimulate, but this exaltation is followed by a corresponding stage of depression, and thus the nervous system is gradually undermined. Opium long continued deranges the whole system; and by deranging the digestion, through its influence on the nervous system and the secretions, comes in conflict with the principal object of treatment, namely: support.

He substitutes food, moderate, occasional stimulation, and avoids perturbation of any kind whatever.

Dr. John G. Westmoreland replied, that if opium was given for a short time and then suspended, that depression would result; but he does not suspend it until convalesence is declared. He continues its use to keep the patient from dying till recovery can take place.

Dr. Raushenberg does not regard opium as a direct stimulant to the brain. When atoxic symptoms and restlessness, with a small, hard, frequent pulse, obtains, he thinks opium is demanded; but where there is general stupor, opium is contra indicated. Opium stimulates the action of the heart, but increases depression when it exists. Alcohol, on the other hand, is not only a direct stimulant to the brain, but furnishes a hydrocarbonaceous material.

Dr. John Stainback Wilson gives opium in certain conditions to obtund sensibility; uses cardiac sedatives, generally the veratrum viride; but considers attention to diet the most important object.

Dr. John G. Westmoreland prescribes diet for his patients—the article, character, amount, and the hour at which it is to be taken. He thinks that opium increases the power to digest.

Dr. W. F. Westmoreland used alcoholic stimulants in the treatment of typhoid fever twenty years ago. He took the position then that it was a prophylactic against typhoid fever, and demonstrated it to his satisfaction. He gives it not only as a stimulant, but to neutralize the poison in the blood. He relies almost exclusively on alcohol and diet. Objects to the constant administration of opium, but certain irritable cases demand it. Cases resembling congestion, or even inflammation of the brain, after taking two or three grains of opium in as many hours obtain a quiet, refreshing sleep, and awake rational.

Dr. John G. Westmoreland thinks that it is reasonable to suppose that alcohol may act in typhoid as quinine does in malarial fevers. By stimulating a disturbed nervous center it may ward off an attack, but he does not believe that it neutralizes the poison.

Dr. Raushenberg thinks alcohol is antiseptic and may correct changes in the blood. Defibrinized blood cannot stimulate or nourish the nervous system. He holds that the primary lesion is in the blood. Keep the blood up and the nerve power will be sustained. When the temperature is at 102° or 103° F. combustion is very rapid. The amount of urea in the urine indicates active chemical changes. The main value of alcohol is to furnish a hydro-carbon and so save the tissues from destruction. To check the high temperature, cold water, quinine and veratrum viride are the most valuable agents. Irritability and stupor are both due to the poor condition of the blood. It fails to furnish a sufficient amount of nutriment to the nervous centers.

OCTOBER 28, 1872.

The President, Dr. Logan, presiding.

The President reported the following case:

Six weeks ago he was called to see a child eighteen months of age. The mother reported it to be a relapse from pneumonia. She stated that, six weeks before, when it was first attacked, the physician who saw it pronounced it a case of congestion of the lungs. The child seemed to have considerable difficulty about the chest—breathing bad, fever, and a harrassing cough. After ten days the symptoms subsided, and the physician in attendance discharged the case. The child continued feeble, and symptoms of inflammatory action supervening after several days he, (Dr. Logan,) being the family physician, upon his return, after an absence from the city, was called to see it, and pronounced it pneumonia. The patient was treated with veratrum, etc., and a blister to the chest. He continued to improve and relapse for four or five weeks, when, during a violent fit of coughing, a cantaloupe seed was expelled, having an extremely offensive odor. The mother then recollected that the child had had access to some cantaloupe seed. The outer layer of the seed, by long maceration, had become softened and separated from the pulp. Thinks its presence occasioned bronchitis and pneumonia, and that an abscess formed around the seed, as the breath was very offensive for a number of days before it was expectorated. A seed similar to the one expectorated was presented for inspection. It was nine weeks ago since the first symptoms appeared, and four or five days since the seed was coughed up. The child rapidly improved for some days, and gave strong hopes of recovery, but finally died.

Dr. John G. Westmoreland reported a case recently seen by him. A young man, first seen in the afternoon, had been sick for twenty-four hours. Pulse 120; sweating profusely; no other prominent symptom. The patient was subject to attacks of intermittent fever, and this was diagnosed to be such, and quinine prescribed. Was called to see him again next morning. He had taken twenty or thirty grains of quinine, yet the fever continued; had an irritative cough; breathing seventy per minute; right lung dull on percussion; crepitant rale present; expectorating mucus; no blood. Was the engorgement, effusion, or inflammation, dependent on malaria? The absence of blood in the expectorating was remarkable. The patient died sixty hours after the attack.

Dr. W. F. Westmoreland remarked that blood was not necessarily present in the expectoration of pneumonia. When the lung is rapidly hepatized, no blood is found in the expectoration.

Dr. W. F. Westmoreland called attention to and condemned the practice advocated by some gentlemen, at a meeting of the Academy a few weeks ago, of leaving the placenta to take care of itself in cases of abortion, after the expulsion of the fœtus. He considers it a dangerous practice.

Dr. John G. Westmoreland advocated that treatment at the meeting referred to, and would respond. Occasionally, in his experience, the placenta has been expelled with the fœtus; but in most cases is retained for several days. The first case of abortion that he ever treated gave him some uneasiness; he attempted to remove the placenta, but could not, so was compelled to allow it to remain, and no harm resulted. Has never had a case of dangerous hemorrhage from retained placenta in abortion. Recently had a case at three months; scarcely any hemorrhage—not more than the lochial discharge at full term—and as usual the placenta came away four or five days after the expulsion of the fœtus. He lets them remain because he can not help himself.

Dr. W. F. Westmoreland was surprised that during so many years of practice the gentleman had never seen a case of flooding from retained placenta. His experience is certainly a very different one. The cases which have alarmed him most in obstetrics, were hemorrhages from retained placenta after abortion. A few months ago, a lady aged forty-five years, in this city, aborted. Twenty-four hours after, the placenta was partially expelled, with great hemorrhage. He found the woman almost pulseless and the hemorrhage continuing. Has tried ergot, gossypium, etc., with no benefit. He removes the placenta to avoid the risk of hemorrhage and septic decomposition of the placental mass. Has never experienced great difficulty in removing; can bear down the uterus and introduce a finger into the os, generally without difficulty. He would not hesitate to dilate by means of a sponge tent, if necessary. Several years ago saw a lady in consultation. No effort had been made at the time of the expulsion of the fœtus to remove the placenta; several days had elapsed, and she had frequent hemorrhages; she was almost bloodless. He introduced his finger and removed the placenta, and notwithstanding the free use of ergot, it was necessary to introduce the finger into the uterus from time to time, for several hours, to induce contraction.

The President concurs with Dr. W. F. Westmoreland. Has seen frequent hemorrhages. Is always apprehensive until the placenta is expelled. His invariable plan is to introduce the tampon in all cases under the third month. Regards the patient then as perfectly safe; a dangerous amount of hemorrhage is impossible. Removes the tampon after twelve or twenty-four hours; and as a rule finds the placenta in the vagina. The presence of the tampon also stimulates the uterus to contraction.

Dr. John G. Westmoreland advocated the use of the tampon, or an attempt to remove the placenta if hemorrhage should occur. Thinks his experience proves that the danger from hemorrhage in such cases is over-estimated.

Dr. Rauschenberg.—Dr. John G. Westmoreland's experience is certainly rare. He has seen most frightful hemorrhages in this class of cases, and obstetric literature confirms the views of Drs. W. F. Westmoreland and Logan. Thinks the best practice is, just as soon as the hemorrhage occurs, to remove the placenta, if possible; if not, tampon. Has used lately a sponge tent, and after eight or nine hours, when the tampon was removed, the tent and placenta were both found in the vagina.

The President remarked, that in many cases the most serious part of the hemorrhage occurs when the fœtus is expelled, as the placenta is often simultaneously separated, and no hemorrhage occurs after this separation takes place, even though the placenta is retained in the cavity of the uterus.

